# Utilization of CT and MRI scanning in Taiwan, 2000–2017

**DOI:** 10.1186/s13244-023-01364-2

**Published:** 2023-02-03

**Authors:** Chung-Chien Huang, Fransisca Fortunata Effendi, Russell Oliver Kosik, Wen-Jeng Lee, Li-Jen Wang, Chun-Jung Juan, Wing P. Chan

**Affiliations:** 1 International Ph.D. Program In BioTech And Healthcare Management, Department of Health Care Administration, College of Management, Taipei Medical University, Taipei, Taiwan; 2 Department of Medical Quality, Wan Fang Hospital, Taipei Medical University, Taipei, Taiwan; 3 Department of Radiology, Wan Fang Hospital, Taipei Medical University, 111 Hsing Long Road, Section 3, Taipei, Taiwan; 4 Department of Radiology, School of Medicine, College of Medicine, Taipei Medical University, Taipei, Taiwan; 5 Department of Medical Imaging, National Taiwan University Hospital, Taipei, Taiwan; 6 Department of Medical Imaging, National Taiwan University College of Medicine, Taipei, Taiwan; 7 Department of Medical Imaging and Intervention, New Taipei Municipal TuCheng Hospital, Chang Gung Medical Foundation, Taoyüan, Taiwan; 8 Department of Medical Imaging and Intervention, Linkou Chang Gung Memorial Hospital, College of Medicine, Chang Gung University, Linkou, Taiwan; 9 Department of Medical Imaging, China Medical University Hsinchu Hospital, Hsinchu City, Taiwan; 10 Department of Radiology, School of Medicine, China Medical University, Taichung, Taiwan

**Keywords:** Computed tomography, Diagnostic imaging, Magnetic resonance imaging, Epidemiology, Utilization

## Abstract

**Objectives:**

This population-based study aimed to collect, analyze, and summarize the long-term trends in medical imaging use in Taiwan.

**Methods:**

A retrospective cohort population-based study of medical imaging usage for the individuals who received care under the National Health Insurance system from 2000 to 2017. CT and MRI utilization rates were determined overall as well as across certain variables including patient age, hospital type, health care type, hospital characteristics, and geographic area.

**Results:**

Individuals registered in our health insurance system have received 21,766,745 CT scans and 7,520,088 MRI scans from 2000 to 2017. Annual growth rates for both imaging types were positive over that period, though growth rates have slowed in recent years. The growth rate for CT use was greatest (9–12%) between 2001 and 2004, dropped to 2% in 2005, then generally rose thereafter, reaching 3% in 2017. Similarly, MRI use growth peaked at 24% between 2001 and 2003, dropped to 4% in 2005, then increased in a fluctuating manner, reaching 2% in 2017.

**Conclusion:**

Over the past 2 decades, CT and MRI use in Taiwan has increased sharply, especially in the oldest age group (≥ 60 years old), but growth rates have slowed in recent years. Increases in imaging use have corresponded with improved clinical outcomes, including greater life expectancy and reduced mortality rates, though further assessment is required to demonstrate a direct link with imaging. Nevertheless, the better clinical outcomes are also predisposed by the comprehensive care covered by the NHI system.

**Supplementary Information:**

The online version contains supplementary material available at 10.1186/s13244-023-01364-2.

## Key points

1. CT and MRI use in Taiwan has elevated prominently in the two past decades, particularly in the elderly adults (≥ 60 years old).
2. The growth rates have moved to a slower pace in recent years.
3. The escalation of medical imaging utilization has been correlated with elevated clinical outcomes.

**Supplementary Information:**

The online version contains supplementary material available at 10.1186/s13244-023-01364-2.

## Introduction

The utilization of medical imaging technologies, including computed tomography (CT) and magnetic resonance imaging (MRI), has increased dramatically in recent years [1]. These trends have been observed in developed countries, including the United States [2], European countries [3–5], and Japan [6]. CT use in Taiwan steadily rose between 2000 and 2013, from 24,257 to 60,351 scans per million people per year, an average yearly increase of 7.4 ± 5.9% [7].

Dramatic growth in health care costs is a challenge faced by all countries. The primary causes include aging populations (as one of the burden of cancer disease [8]), an increasing number of patients with chronic diseases, and the availability of new, advanced medical technologies [9]. This has driven the demand for diagnostic tests that can improve outcomes through early diagnosis. In March 1995, Taiwan established the National Health Insurance (NHI) program, which covers 99.8% of Taiwan’s population. This government-based system was founded on a single-payer model [10]. Taiwan’s health policies have enabled the public to access and afford medical imaging services through a nation-wide equitable system [11]. However, few studies have examined medical imaging utilization in Taiwan.

In this study, we aim to scrutinize long-term trends in high-end medical imaging utilization in Taiwan. We also aim to evaluate the changes in medical imaging usage that have occurred over time across certain patient demographic factors, hospital levels, health care types, and other characteristics.

## Methods

CT and MRI usage data were collected from the NHI system in Taiwan for the period 2000 through 2017. Because a large majority of the Taiwanese population (± 23.57 million people) is covered under this system, this data is representative of Taiwan’s health care system overall, and it adequately reflects the patient population in terms of sociodemographics, geographic diversity, and hospital characteristics.

### Data source

Data from 2000 through 2017 were obtained from Taiwan’s National Health Insurance Research Database (NHIRD), which is compiled by the Statistics Department at the Ministry of Health and Welfare. The NHIRD contains comprehensive health records that have been integrated into the NHI Cloud-Based Inquiry System for Medical Care Information, which permits sharing of medical histories, medication records, and medical imaging across all health care facilities and settings (inpatient, outpatient, and emergency departments [ED]), with a long-term goal of improving patient safety [10]. Taiwan classifies hospitals as either district hospitals, regional hospitals, or medical centers. At the top of the medical care system are the medical centers, each with at least 500 beds (at least 25 for acute mental illness) and a highly specialized staff (e.g., internal medicine, general surgery, neurosurgery, plastic surgery, and specialized imaging units) [12]. Meanwhile, regional hospitals, have at least 250 beds, and district hospitals are the smallest of the three. The cities of Taipei and Kaohsiung are considered Metropolitan Areas [13].

### Imaging utilization

The longitudinal dataset contained details regarding service type, patient diagnosis, prescription information, and cost. All imaging examinations were coded using either the *Current Procedural Terminology; International Classification of Diseases, Ninth Revision, Clinical Modification*; and/or the *International Statistical Classification of Diseases, Tenth Revision, Clinical Modification* as well as by the Healthcare Common Procedure Coding System billing codes, based on the study performed. If medical imaging was used for or integrated with radiotherapy planning, image processing, or reinterpretation, it was not tallied.

The following data points were tabulated: the annual numbers of CT and MRI scans, the annual growth rate (percent change year-over-year), and the cumulative percent change (the number of scans performed in the current year divided by the number performed in 2000). Usage rates by age were determined for 10 year age intervals through age 79 years (0–9 years, 10–19 years, etc.) and for those aged 80 years or more.

The numbers of CT and MRI scans performed across different hospital levels, health care types, and certain hospital characteristics were also tabulated. Hospital levels included either medical center, regional hospital, or district hospital. Health care types included either outpatient, emergency, or inpatient. Hospital characteristics included type (private versus public and teaching versus non-teaching) and location (Northern [Taipei City] and Southern [Kaohsiung and Pingtung] metropolitan areas compared to other areas North District excluding Taipei, Central District, South District excluding Kaohsiung and Pingtung, and East District).

### Calculations

#### Age-standardized use rate

The age-standardized usage rate was determined by multiplying the crude utilization rate by the world population per the WHO (2000) [14] and dividing by 100,000 people.

$${\text{Crude}}\;{\text{utilization}}\;{\text{rate}} = \frac{{{\text{Total}}\;{\text{number}}\;{\text{of}}\;{\text{CT/MRI}}\;{\text{usage}}\;{\text{per}}\;{\text{each}}\;{\text{age}}\;{\text{group}}}}{{{\text{Total}}\;{\text{population}}\;{\text{per}}\;{\text{each}}\;{\text{age}}\;{\text{group}}}} \times 10^{5}$$

$${\text{Age}} - {\text{standardized}}\;{\text{usage}}\;{\text{rate}} = \frac{{{\text{Crude}}\;{\text{utilization}}\;{\text{rate}} \times {\text{world}}\;{\text{population}}\;{\text{by}}\;{\text{WHO}}\left( {2000} \right)}}{{10^{5} }}$$

#### Annual growth rate

The CT and MRI annual growth rates were calculated using the following formula:

$${\text{AGR}} = \frac{B}{A} - 1$$

where *B* is the number of CTs or MRIs performed in a given year, and *A* is the number performed in the prior year [1]. For example, the annual growth rate for 2017 was calculated based on the increase (or decrease) in the number of scans performed in 2017 compared to 2016 divided by the number of scans performed in 2016.

### Statistical analyses

Study measures were quantified based on the statistical method used to perform the descriptive analysis (i.e., rate, percentage, and growth rate). All analyses were performed for each imaging modality by age group, hospital level, hospital type, geography, and health care type. For each modality, we calculated the imaging rate per 1000 persons per year. The analyses were performed using SAS statistical software, version 9.4 (SAS Institute Inc).

## Results

**Fig. 1:**
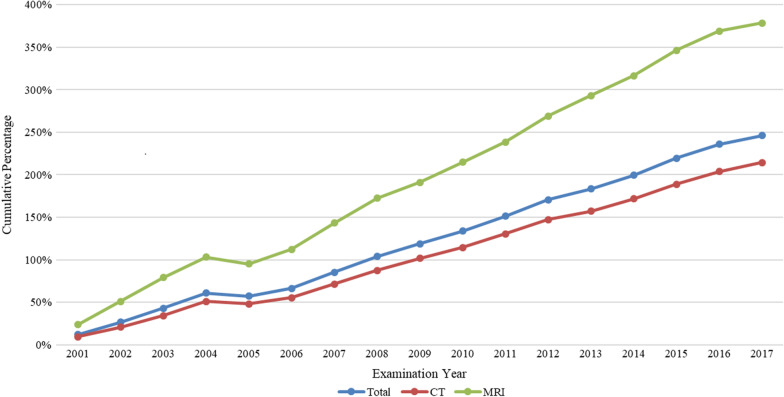
The cumulative percentage of medical imaging utilization in Taiwan from 2000 to 2017

The long-term data indicate that CT and MRI use has generally rose year-over-year. The total number of CT and MRI scans performed from 2000 through 2017 was 29,286,833 (35,671 scans with missing data and 22 scans performed elsewhere were excluded). Of these, 21,766,745 (an annual average of 1,209,264) were CTs and 7,520,088 (an annual average of 417,783) were MRIs. In total, 748,197 scans were performed in 2000 and 2,590,370 scans were performed in 2017, an overall increase of 246%. By imaging modality, the number of annual CTs and MRIs performed increased 215% and 379%, respectively (Table 1 and Fig. 1).

**Table 1:** Medical imaging utilization in Taiwan from 2000 to 2017

Characteristics	Overall no.	Computed tomography	Magnetic resonance imaging
*Total No. of tests per 1000 person-years*			
2000	34	27	6
2001	37	29	8
2004	53	40	13
2005	52	39	12
2008	66	49	17
2009	71	53	18
2012	87	64	23
2013	91	66	24
2016	107	78	29
2017	110	81	29
*Growth rate (in percentage)*			
2001	12	9	24
2005	− 2	− 2	− 4
2009	7	8	7
2013	5	4	7
2017	3	3	2
*Cumulative percentage (in percentage)*			
2001	12	9	24
2005	57	48	95
2009	119	102	191
2013	184	157	293
2017	246	215	379

**Fig. 2:**
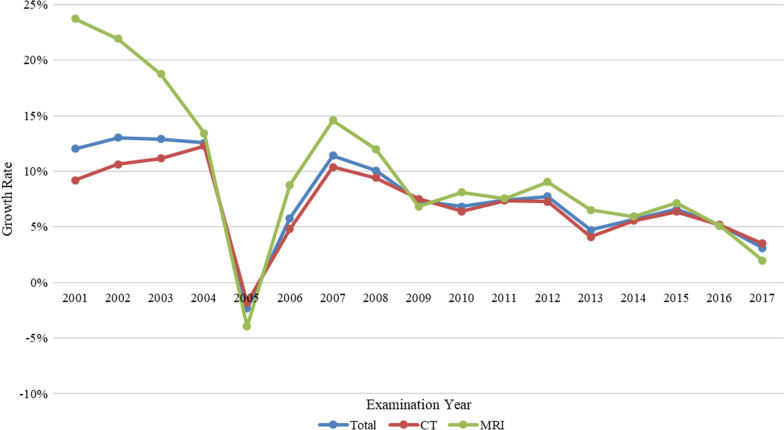
The growth rate of medical imaging utilization in Taiwan from 2000 to 2017

**Fig. 3:**
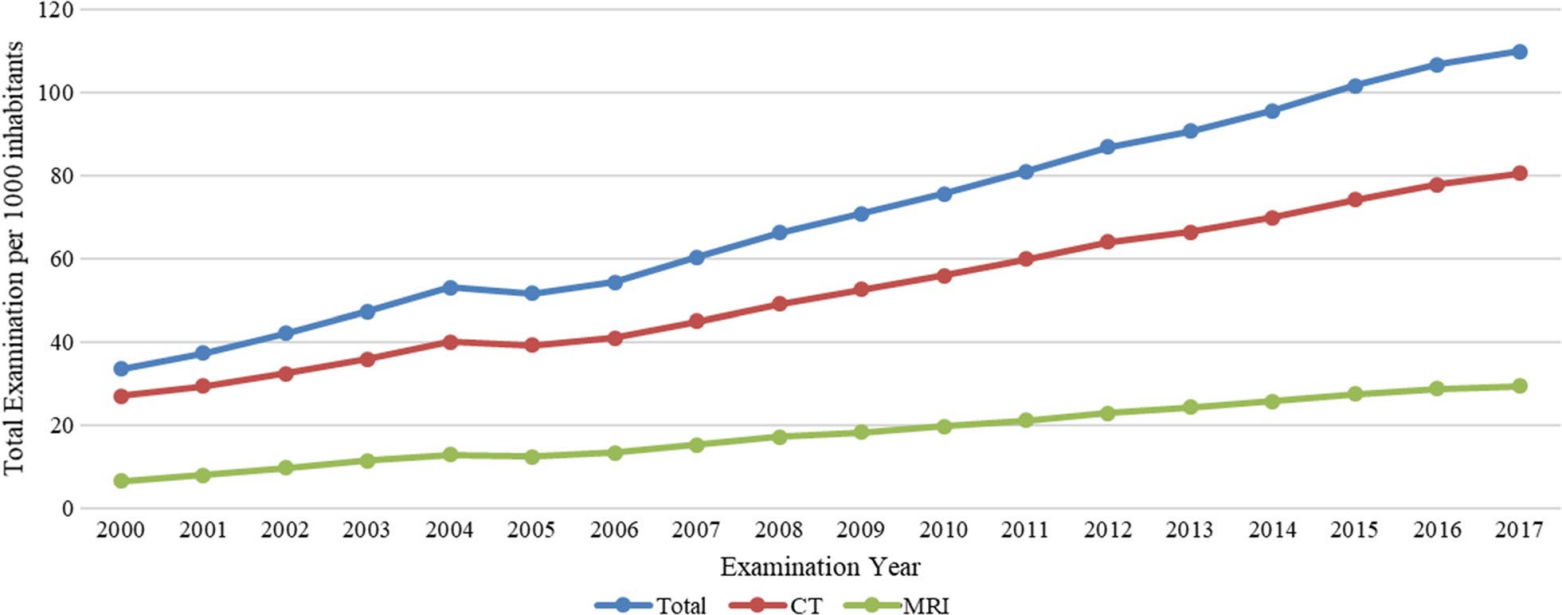
Annual numbers of medical imaging examinations performed in Taiwan from 2000 to 2017 (per 1000 inhabitants)

CT and MRI annual growth rates peaked in the earlier years (between 2001 and 2004), and overall annual growth patterns were similar between these modalities. Both showed strong year-over-year growth rates during these earlier years (for CT, growth increased from 9 to 12%), however MRI growth decreased over this period, from 23 to 13%. Usage dropped in 2005, but growth began again in 2006, reaching 10% for CTs and 15% for MRIs. In 2017, the year-over-year growth rates for CT and MRI were 3% and 2%, respectively (Table 1 and Fig. 2). The usage rates for CT imaging increased from 27 per 1000 person-years in 2000 to 81 per 1000 person-years in 2017. For MRI, usage rates increased from 6 per 1000 person-years in 2000 to 29 per 1000 person-years in 2017 (Table 1 and Fig. 3).

### Comparison across age groups

In general, upward trends in utilization were seen for both modalities across all age groups, but older individuals accounted for 54% of the overall imaging (Table 2), including 52% of the total CT use and 44% of the total MRI use (Additional file 1: Table S1). The high usage rates in older individuals (1134 per 100,000 people) was however expected, given the increased prevalence and complexity of diseases as people age.

**Table 2:** Use of CT and MRI by Age Group per 100,000 Population in 2017^a^

Age group, y	Population	Scans	%	Crude utilization rate (per 10^5^)	Age-standardized usage rate	WHO 2000, f
0–9	2,024,831	22,266	0.01	1100	192	17,500
10–19	2,425,114	54,113	0.02	2231	382	17,100
20–29	3,223,754	122,346	0.05	3795	611	16,100
30–39	3,732,974	186,146	0.07	4987	738	14,800
40–49	3,680,233	30,218	0.12	8185	1031	12,600
50–59	3,634,503	484,859	0.19	13,340	1334	10,000
60–69	2,803,961	590,802	0.23	21,070	1412	6700
70–79	1,278,868	441,094	0.17	34,491	1276	3700
≥ 80	766,989	365,717	0.14	47,682	715	1500
Missing	–	21,809	0.01	–	–	–
Total	23,571,227	2,590,370	1	10,990	–	100,000

### Comparison across health care types

From 2000 to 2017 (Tables 3 and 4), the greatest number of CTs came from the outpatient setting (514,357 CTs or 22 per 1000 persons per year (43%)), followed by the emergency setting [361,576 CTs or 16 per 1000 persons per year (30%)], and finally by the inpatient setting [333,331 CTs or 14 per 1000 persons per year (28%)]. The greatest number of MRIs also came from the outpatient setting [311,268 MRIs or 13 per 1000 persons per year (75%)], followed by the inpatient setting [96,242 MRIs or 4 per 1000 persons per year (23%)], and finally by the emergency setting [10,273 MRIs or 0.4 per 1000 persons per year (2%)].

**Table 3:** Average Annual CT and MRI Utilization in Taiwan

Characteristics	Hospital level				Metropolitan areas		Other areas				Teaching/non-teaching		Public/private	
	Overall No. (%)	Medical center (%)	Regional hospital (%)	District hospital (%)	Taipei (%)	Kaohsiung–Pingtung (%)	North district (%)	Central district (%)	South district (%)	East district (%)	Teaching (%)	Non-teaching (%)	Public (%)	Private (%)
*Computed tomography*														
Inpatient	333,331 (28)	128,268 (11)	153,911 (13)	51,152 (4)	87,772 (7)	80,088 (7)	41,877 (3)	52,426 (4)	62,590 (5)	8579 (0.7)	301,000 (25)	32,331 (3)	112,220 (9)	221,111 (18)
Emergency	361,576 (30)	143,505 (12)	172,528 (14)	45,544 (4)	128,404 (11)	49,503 (4)	54,951 (5)	71,355 (6)	46,043 (4)	11,319 (0.9)	336,361 (28)	25,215 (2)	100,563 (8)	261,014 (22)
Outpatient	514,357 (43)	252,564 (21)	198,451 (16)	63,342 (5)	193,801 (16)	75,326 (6)	66,386 (5)	95,435 (8)	71,493 (6)	11,916 (0.99)	472,814 (39)	41,543 (3)	178,830 (15)	335,526 (28)
*Magnetic resonance imaging*														
Inpatient	96,242 (23)	49,742 (12)	42,677 (10)	3823 (0.9)	33,945 (8)	18,749 (4)	10,214 (2)	18,150 (4)	12,987 (3)	2197 (0.5)	94,097 (23)	2146 (0.5)	29,387 (7)	66,855 (16)
Emergency	10,273 (2)	7055 (2)	2838 (0.7)	380 (0.1)	4559 (1)	664 (0.2)	1013 (0.2)	1770 (0.4)	1331 (0.3)	936 (0.2)	10,057 (2)	216 (0.05)	3727 (0.9)	6545 (2)
Outpatient	311,268 (75)	141,789 (34)	139,941 (33)	29,538 (7)	123,572 (30)	38,842 (9)	35,743 (9)	65,154 (16)	39,851 (10)	8105 (1.9)	291,227 (70)	20,040 (5)	96,214 (23)	215,053 (51)

*CT* Computed tomography; *MRI* Magnetic resonance imaging

**Table 4:** Average Annual CT and MRI Utilization in Taiwan (per 1000 Inhabitants)

Characteristics		Hospital level			Metropolitan areas		Other areas				Teaching/non-teaching		Public/private	
	Overall	Medical center	Regional hospital	District hospital	Taipei	Kaohsiung–Pingtung	North district	Central district	South district	East district	Teaching	Non-teaching	Public	Private
*Computed tomography*														
Inpatient	14.4	5.6	6.7	2.2	3.8	3.5	1.8	2.3	2.7	0.4	13.0	1.4	4.9	10
Emergency	15.6	6.2	7.4	2.0	5.5	2.1	2.4	3.1	2.0	0.5	14.5	1.1	4.3	11
Outpatient	22.2	10.9	8.6	2.7	8.4	3.3	2.9	4.1	3.1	0.5	20.4	1.8	7.7	14
*Magnetic resonance imaging*														
Inpatient	4.2	2.2	1.8	0.2	1.5	0.8	0.4	0.8	0.6	0.1	4.1	0.1	1.3	3
Emergency	0.4	0.3	0.1	0.02	0.2	0.03	0.04	0.1	0.1	0.04	0.4	0.01	0.2	0.3
Outpatient	13.4	6.1	6.0	1.3	5.3	1.7	1.5	2.8	1.7	0.4	12.6	0.9	4.1	9

*CT* Computed tomography; *MRI* Magnetic resonance imaging

### Comparison across specialty departments

Emergency departments ordered CTs most frequently (32%) followed by neurology (12%) and thoracic medicine (11%) departments. In contrast, neurosurgery departments ordered MRIs most frequently (29%) followed closely by orthopedic (26%) and neurology (22%) departments.

### Comparison across hospital levels

Imaging use was comparable at medical centers and regional hospitals, but occurred somewhat less frequently at district hospitals. Regional hospitals [77 of 451 hospitals (17%) in 2017] [18] ordered the most CTs [524,889 CTs or 23 per 1000 persons per year (43.41%)], followed by medical centers [22 of 451 hospitals (5%) in 2017] [15] [524,337 CTs or 23 per 1000 persons per year (43.36%)], and finally by district hospitals [307 of 451 hospitals (68%) in 2017] [15] [160,038 CTs or 7 per 1000 persons per year (13%)]. In contrast, medical centers accounted for the largest share of MRIs [198,587 MRIs or 9 per 1000 persons per year (48%)], followed by regional hospitals [185,456 MRIs or 8 per 1000 persons per year (44%)], and finally by district hospitals [33,740 MRIs or 1 per 1000 persons per year (8%)].

### Comparison across hospital types

Despite non-teaching hospitals [316 of 451 hospitals (70%) in 2017] [15] greatly outnumbering teaching hospitals, teaching hospitals ordered the majority of both CTs and MRIs (92% and 95%, respectively), accounting for 1,110,175 CTs or 48 per 1000 persons per year and 395,381 MRIs or 17 per 1000 persons per year. Similarly, private hospitals [403 of 483 hospitals (83%) in 2017] [15] ordered the majority of both CTs and MRIs (68% and 69%, respectively) accounting for 817,651 CTs or 35 per 1000 persons per year and 288,454 MRIs or 12 per 1000 persons per year.

### Comparison across geographic areas

Hospitals in metropolitan areas [144 of 483 hospitals (30%) in 2017] [15] used imaging slightly more frequently than hospitals in other areas, averaging 614,894 CTs or 27 per 1000 persons per year (51% of all CTs) and 220,332 MRIs or 10 per 1000 persons per year (52% of all MRIs). Usage in other areas however did not lag significantly, averaging 594,370 CTs or 26 per 1000 persons per year and 197,450 MRIs or 9 per 1000 persons per year.

## Discussion

In this study, we assess the imaging growth rates that occurred from the year 2000 forward. While growth rates initially accelerated sharply, a significant drop-off occurred in 2004 and 2005. This is almost certainly a sequela of the severe acute respiratory syndrome (SARS) outbreak that hit Taiwan in 2003 and 2004 [16, 17]. Patients with non-emergent or less severe diseases likely felt anxious about visiting hospitals and clinics, resulting in a reduction in the use of medical imaging services [11]. The number of scans performed in 2005, the second year of the SARS outbreak, returned to pre-SARS levels only in terms of ED imaging (Additional file 1: Table S2). This likely reflects the increased need for emergent imaging related to the SARS outbreak.

The rates of CT and MRI usage were appreciably greater in the older population (54% of all scans performed in 2017), paralleling the growth of that population that has occurred in Taiwan (74.05% over the past 17 years). Specifically, the three most frequent categories of users of CT and MRI services were all older individuals who received the services as outpatients (Table 2). Likewise, outpatients accounted for a large proportion of the older population. Smith-Bindman reported similar growth rates for CT use in the older population (aged ≧ 65 years) in the United States, which reached 9.5% from 2000 to 2006, and was therefore similar to the growth rate seen in Ontario, Canada, from 2000 to 2007 [5]. After slowing between 2006 and 2013, the CT use growth rate in the older population in the US accelerated again, reaching 5.2%, from 2014 to 2016. Comparatively, MRI growth rates in the older population were 11.3% in the United States (2000–2005) and 22% in Ontario (2000–2007), slowing thereafter to 2.2% (2005–2016) and 4.9% (2010–2016), respectively [5]. In terms of other Asian countries, aside from South Korea, the Taiwanese elderly population has grown the fastest (109.12% versus 74.05%). The elderly populations in New Zealand, Australia, and Japan have also risen considerably (66.89%, 62.29%, and 56.75%, respectively). In addition, many other developed countries have seen growth rates in their elderly populations of between 25 and 50% over the past two decades (Additional file 1: Table S3) [18–21].

In 2017, approximately 61.5% of the CT scanners in Taiwan were 64 or greater-slice models, allowing for image quality and scanning speeds much greater than those derived from the original 4-slice scanners. In 2015, this figure was 50.40% [22]. Similarly, modern 3-T MRI systems are becoming more prevalent [23], accounting for 14.29% of all systems in 2015 and 15.93% in 2017 [24]. Therefore, in addition to physician and patient demand, easy access, and financial incentives (supportive policies by the Taiwanese government), technical improvements have also contributed to the rapid rise in CT and MRI usage.

Our results reveal that medical centers order almost equivalent numbers of CTs to those of regional hospitals and MRIs more than regional and local hospitals, suggesting that they should be if they are not already equipped with comparatively more advanced technologies. Given the generally greater frequency of complicated diseases that medical centers treat, a higher level of imaging quality is necessary [25].

Hospitals in the northern and southern metropolitan areas tended to have more equipment and resources, likely explaining their slightly increased utilization rates. However, the nearly similar rates seen in other areas are a testament to the egalitarianism of the health care system in Taiwan, despite the fact that metropolitan areas tend to have larger populations [25].

Despite a relatively low use of imaging (80.5 CTs and 29.4 MRIs per 1000 population in 2017) compared to other countries (Additional file 1: Table S4), Taiwan’s life expectancy has increased from 75.9 years in 2000 to 80.9 years in 2018, a rate comparable to those in other developed countries (Japan, 84.2 years; Korea, 82.7 years; and the United Kingdom, 81.3 years) and exceeding that in the United States (78.7 years) (Additional file 1: Table S5) [26]. However, global mortality due to malignant neoplasm is rapidly rising in the older population worldwide [27, 28], and Taiwan is not likely to be spared in the future. The diagnosis and treatment of malignancies requires extensive imaging and is likely to contribute to growth rates in imaging going forward.

Across Taiwan, there were 379 CT and 210 MRI scanners in 2015. This increased to 408 and 226, respectively, in 2017 [22, 24]. Worldwide, Japan had the most scanners per 1,000,000 inhabitants in 2017 (111.5 CT and 55.2 MRI scanners) followed by Australia (64.3 CT scanners) and the United States (39.2 MRI scanners). Taiwan lagged these countries considerably, with 16.9 CT scanners and 10.0 MRI scanners (Additional file 1: Table S6) [29, 30]. Therefore, Taiwan proves that high life expectancy can still exist despite a relatively low availability of CT and MRI scanners.

Enrollment in the NHI plan is mandatory for all citizens and foreign residents of Taiwan. NHI covers up to 99.8% of Taiwan’s 23.57 million inhabitants. It ensures that every resident has access to quality and affordable medical care, providing comprehensive coverage for emergency, inpatient, and outpatient care. In the United States, total estimated annual wasted spending on healthcare in 2019 has ranged from US $760 billion to US $935 billion. With total health care spending in 2019 projected at approximately US $3.82 trillion, it is believed that about 25% of these costs can be eliminated [31]. In Taiwan, wasted spending on healthcare amounted to more than US $55.55 million in 2017, driven in part by patients lost to follow-up after CT or MRI imaging, and ultimately leading some to repeat the tests at other hospitals [25]. According to data from 2004 and 2005, 21.5% of such patients returned within 90 days for a repeat scan. Repeat CTs and MRIs are performed most often on patients with malignancies (31.8%), neurologic disorders (24.0%), and brain or spinal injuries (25.3%) [39]. In 2019, the NHI restricted repeat scanning within 28 days without appropriate clinical indications [32].

Given the significant growth, medical imaging in Taiwan has taken up a larger and larger portion of the NHI budget in recent years [33]. Its annual expenditure totaled only US $ 8.9 billion in 2000 though reached US $19.9 billion in 2017 [34]. Some have questioned the cost-effectiveness of increased imaging, particularly in the emergency setting. Because most ED visits (48.0%) are made by older patients with multiple clinical problems, classifying those visits based on symptoms has proven difficult. However, Taiwan has observed a decline in admission rates following emergency CT, which was 59.9% in 2009 and only 48.2% in 2013 [35]. This suggests that there has been an increase in the number of unnecessary emergency scans performed. Thus, NHIA presented the patient referral system to lower down the outpatient volume at larger hospitals and set them to focus on providing the treatment for the illness. As a result, patient without referral requires to pay higher out-of-pocket fees. In addition, the cloud-based data sharing system introduced by NHIA have already reduced the amount of medical examinations performed (CT scans, in specific) and saved USD 38.86 million for 6 months of the second semester in 2017 [36].

Future imaging rates in Taiwan are likely to be driven by two opposing factors: population decline and aging. Declines in birth rates, and therefore overall population, is expected to drive imaging rates lower, particularly in the long run. However, aging is expected to increase imaging rates in the short term, given that older people tend to require more imaging [37]. Moreover, the diagnostic of patient with metastatic diseases has also rose up the usage of medical imaging, especially in the last month of their lives [38], which was in-line with the escalating cases of its incidence rate [39]. The cumulative effect on the number of imaging studies performed is difficult to predict, but imaging per capita is likely to increase.

The major strength of this study is that the data includes all CTs and MRIs performed within the NHI system in Taiwan, which essentially includes the entire population. In addition, because the number of scans performed was taken from a single-payer billing database (in contrast to self-reported survey data or data obtained from the literature), the data is likely to be highly accurate.

### Limitations

Some patients had more than one anatomical area scanned during a single visit were recorded as having received only a single scan. In addition, self-payment imaging was not included. These likely resulted in a slight underestimate of the total number of scans performed. Furthermore, this study only analyzed the numbers of uses, not adjusting to the individuals. Also, detailed information on the diseases of which imaging was performed could not be included, so the classification of diseases was challenging. This highlights the need for further large-scale studies for the justifications of imaging conducted and evaluation of imaging usage in the particular diseases.

## Conclusion

Over the past 2 decades, CT and MRI use in Taiwan has increased sharply, especially in the oldest age group (≥ 60 years old), but growth rates have slowed in recent years. Increases in imaging use have corresponded with improved clinical outcomes, including greater life expectancy and reduced mortality rates, though further assessment is required to demonstrate a direct link with imaging. Nevertheless, the better clinical outcomes are also predisposed by the comprehensive care covered by the NHI system.

### Supplementary Information

**Additional file 1: eTable 1.** Demographic Data of the Sample Population. **eTable 2.** The Volumes of the Outpatient, Inpatient and Emergency Services Before, During and After the SARS Epidemic. **eTable 3.** Comparison of Elderly Population (over 65 years old) in Selected Countries (in alphabetical order). **eTable 4.** Number of CT and MRI Scanners in Selected Countries per 1,000,000 Inhabitants (in alphabetical order). **eTable 5.** Life Expectancy and Mortality Rates in Selected Countries (in Alphabetical Order). **eTable 6.** Number of CT and MRI Scanners in Selected Countries per 1,000,000 Inhabitants (in alphabetical order).

## Data Availability

The datasets generated and analyzed during the current study are not publicly available due to the policy of the Research Ethics Committee of Taipei Medical University—Joint Institutional Review Board but are available from the corresponding author on reasonable request.

## Abbreviations

CT: Computed tomography
MRI: Magnetic resonance imaging
