# Supplementary material for: Utilization of CT and MRI scanning in Taiwan, 2000–2017
Source: Insights Imaging. 2023 Feb 3;14:23. doi: 10.1186/s13244-023-01364-2 (PMC9897145; doi:10.1186/s13244-023-01364-2)
Supplement: Supplementary file 1 — Additional file 1: eTable 1. Demographic Data of the Sample Population. eTable 2. The Volumes of the Outpatient, Inpatient and Emergency Services Before, During and After the SARS Epidemic. eTable 3. Comparison of Elderly Population (over 65 years old) in Selected Countries (in alphabetical order). eTable 4. Number of CT and MRI Scanners in Selected Countries per 1,000,000 Inhabitants (in alphabetical order). eTable 5. Life Expectancy and Mortality Rates in Selected Countries (in Alphabetical Order). eTable 6. Number of CT and MRI Scanners in Selected Countries per 1,000,000 Inhabitants (in alphabetical order). [file 13244_2023_1364_MOESM1_ESM.docx]

Original article

**Utilization of CT and MRI scanning in Taiwan, 2000-2017**

**eTable1.** **Demographic Data of the Sample Population**

| **Characteristics** | **Overall No. (%)** | **Computed Tomography (%)** | **Magnetic Resonance Imaging (%)** |
| --- | --- | --- | --- |
| Total | 29 286 833 | 21 766 745 | 7 520 088 |
| **Age group** |  |  |  |
| Children (≤19 y) | 1 259 431 (4) | 916 112 (4) | 343 319 (5) |
| Adults (20-59 y) | 13 196 219 (43) | 9 367 138 (43) | 3 829 081 (51) |
| Older adults (≥60 y) | 14 615 865 (50) | 11 315 806 (52) | 3 300 059 (44) |
|  |  |  |  |
| **No. of person-years by sex** |  |  |  |
| Male | 16 838 354 (54) | 11 927 627 (55) | 4 910 727 (52) |
| Female | 14 145 128 (45) | 9 671 429 (44) | 4 473 699 (47) |
| Unknown/other | 215 318 (0.7) | 167 689 (0.8) | 47 629 (0.5) |
|  |  |  |  |
| **Hospital level** |  |  |  |
| Medical center | 13 012 631 (44) | 9 438 068 (43) | 3 574 563 (48) |
| Regional hospital | 12 786 198 (44) | 9 447 999 (43) | 3 338 199 (44) |
| District hospital | 3 488 004 (12) | 2 880 678 (13) | 607 326 (8) |
|  |  |  |  |
| **Geographic area** |  |  |  |
| **Metropolitan areas** |  |  |  |
| Taipei | 10 296 955 (35) | 7 379 577 (34) | 2 917 378 (39) |
| Kaoshiung and Pingtung | 4 737 108 (16) | 3 688 506 (17) | 1 048 602 (14) |
| **Other areas** |  |  |  |
| North District | 3 783 294 (13) | 2 937 832 (13) | 845 462 (11) |
| Central District | 5 477 227 (19) | 3 945 889 (18) | 1 531 338 (20) |
| Southern District | 4 217 302 (14) | 3 242 277 (15) | 975 025 (13) |
| Eastern District | 774 947 (3) | 572 664 (3) | 202 283 (3) |
|  |  |  |  |
| **Hospital type** |  |  |  |
| Teaching | 27 099 996 (93) | 19 983 146 (92) | 7 116 850 (95) |
| Non-teaching | 2 186 837 (7) | 1 783 599 (8) | 403 238 (5) |
|  |  |  |  |
| Public | 9 376 953 (32) | 7 049 033 (32) | 2 327 920 (31) |
| Private | 19 909 880 (68) | 14 717 712 (68) | 5 192 168 (69) |

**eTable1. Demographic Data of the Sample Population (continued)**

| **Characteristic** | **Overall No. (%)** | **Computed Tomography (%)** | **Magnetic Resonance Imaging (%)** |
| --- | --- | --- | --- |
| **Health care type** |  |  |  |
| Inpatient | 7 732 320 (26) | 5 999 956 (28) | 1 732 364 (23) |
| Outpatient | 14 861 234 (51) | 9 258 419 (43) | 5 602 815 (75) |
| Emergency | 6 693 279 (23) | 6 508 370 (30) | 184 909 (3) |

^1^The total number of scans added based on age group doesn’t match with the total number of procedures because it was only analyzing the number of examinations performed, not adjusting to the individual level.

**eTable2. The Volumes of the Outpatient, Inpatient and Emergency Services Before, During and After the SARS Epidemic**

| **Year** | **Pre-SARS^2^** | **During SARS (%)3** | | **Post SARS (1^st^ y) (%)** | | **Post SARS (2^nd^ y) (%)** | | | **Growth Rate in 2004-2005 (%)** | |  |
| --- | --- | --- | --- | --- | --- | --- | --- | --- | --- | --- | --- |
| **Computed Tomography** | | | | | | | |  | | | |
| Outpatients | 300 972 | | 325 368 (108) | | 358 442 (119) | | 338 472 (112) | | | -19 970 (-0.06) | |
| Emergency services | 168 573 | | 204 875 (122) | | 205 529 (122) | | 233 462 (138) | | | 27 933 (14) | |
| Inpatients | 259 597 | | 280 278 (108) | | 346 221 (133) | | 321 681 (124) | | | -24 540 (-0.07) | |
| **Magnetic Resonance Imaging** | | | | | | | |  | | | |
| Outpatients | 162 825 | | 190 411 (117) | | 208 580 (128) | | 194 784 (120) | | | -13 796 (-0.07) | |
| Emergency services | 3747 | | 4529 (121) | | 4526 (121) | | 6064 (162) | | | 1538 (34) | |
| Inpatients | 51 678 | | 64 256 (124) | | 80 935 (157) | | 81 549 (158) | | | 614 (0.008) | |

2Pre-SARS period was in 2002 and it was used as the reference volume.

3Percentage was calculated based on the increase (or decrease) in the number of scans performed in the 2003 compared to 2002 divided into the number of scans performed in 2002.

**eTable3. Comparison of Elderly Population (over 65 years old) in Selected Countries (in alphabetical order)^4^**

| **Countries** | **Percentage of Elderly Population (Over 65 Years)** | | |
| --- | --- | --- | --- |
|  | **2000** | **2018** | **Growth Rate (%)** |
| Australia | 12.6% | 15.7% | 62.3% |
| France | 16.0% | 19.8% | 35.2% |
| Germany | 16.9% | 21.5% | 27.9% |
| Japan | 18.0% | 28.1% | 56.8% |
| Korea | 7.5% | 14.3% | 109.1% |
| New Zealand | 11.9% | 15.3% | 66.9% |
| Sweden | 17.3% | 19.9% | 32.8% |
| Taiwan | 8.8% | 14.6% | 74.1% |
| United Kingdom | 15.9% | 18.3% | 29.8% |
| United States | 12.4% | 16.0% | 47.2% |

4Data extracted from the report, ‘Elderly population’, Organization for Economic Co-operation and Development, ‘General situation of population’, National Statistics, Republic of China (Taiwan), and ‘Population ages 65 and above, total’, The World Bank Group

**eTable4. Use of CT and MRI in Selected Countries per 1000 Inhabitants (in Alphabetical Order)^5^**

| **Country** | **CT Use** | | **Outpatient CT Use** | | **Inpatient CT Use** | | **MRI Use** | | **Outpatient MRI Use** | | **Inpatient MRI Use** | | |
| --- | --- | --- | --- | --- | --- | --- | --- | --- | --- | --- | --- | --- | --- |
|  | **2007** | **2017** | **2007** | **2017** | **2007** | **2017** | **2007** | **2017** | **2007** | **2017** | **2007** | **2017** |  |
| Australia | 83.2 | 126.0 | 76.9 | 113.5 | 6.3 | 12.5 | 18.9 | 44.8 | 17.8 | 42.9 | 1.1 | 1.9 |  |
| France | 120.0 | 189.7 | 43.6 | 71.0 | 76.4 | 118.7 | 44.1 | 114.1 | 21.1 | 65.3 | 23.0 | 48.8 |  |
| Germany | 102.0 | 153.2 | 60.1 | 79.8 | 42.0 | 73.4 | 83.8 | 149.2 | 69.3 | 124.6 | 14.5 | 24.6 |  |
| Korea | 74.7 | 204.6 | 7.3 | 17.7 | 67.4 | 186.9 | 13.9 | 36.3 | 0.7 | 1.7 | 13.2 | 34.6 |  |
| Taiwan | 45.2 | 80.5 | 18.5 | 35.8 | 13.9 | 17.5 | 15.4 | 29.4 | 11.1 | 22.5 | 3.9 | 6.1 |  |
| United States | 228.1 | 256.3 | 43.2 | 43.7 | 184.9 | 212.6 | 91.3 | 111.1 | 41.8 | 55.4 | 49.5 | 55.7 |  |

5Data extracted from the Organisation for Economic Co-operation and Development

**eTable5. Life Expectancy and Mortality Rates in Selected Countries (in Alphabetical Order)**

| **Country** | **Life Expectancy, y** | | **Mortality Rate (per 1000 live births)** | | **Standardized Cancer Mortality Rate (per 100 000 population)** | |
| --- | --- | --- | --- | --- | --- | --- |
|  | **2000** | **2018** | **2000** | **2018** | **2000** | **2016** |
| Australia | 79.3 | 82.8 | 5.1 | 3.1 | 221.3 | 179.8 |
| France | 79.2 | 82.8 | 4.4 | 3.7 | 236.9 | 196.8 |
| Germany | 78.2 | 81.0 | 4.4 | 3.3 | 234.8 | 200.3 |
| Japan | 81.2 | 84.2 | 3.3 | 1.8 | 208.0 | 171.5 |
| Korea | 76.0 | 82.7 | 6.4 | 2.8 | 217.2 | 165.2 |
| New Zealand | 78.4 | 81.8 | 6.1 | 4.1 | 245.8 | 212.2 |
| Sweden | 79.7 | 82.6 | 3.4 | 2.2 | 205.5 | 179.6 |
| Taiwan | 75.9 | 80.9 | 6.5 | 4.2 | 141.7 | 202.9 |
| United Kingdom | 77.9 | 81.3 | 5.6 | 3.7 | 309.1 | 274.0 |
| United States | 76.7 | 78.7 | 7.1 | 5.6 | 231.1 | 181.9 |

**eTable6. Number of CT and MRI Scanners in Selected Countries per 1 000 000 Inhabitants (in alphabetical order)^6^**

| **Countries** | **CT** | | **MRI** | |
| --- | --- | --- | --- | --- |
|  | **2008** | **2017** | **2008** | **2017** |
| Australia | 39.1 | 64.3 | 5.7 | 14.2 |
| France | 10.8 | 17.4 | 6.1 | 14.2 |
| Germany | 31.2 | 35.1 | 23.6 | 34.7 |
| Japan | 97.0 | 111.5 | 43.0 | 55.2 |
| Korea | 36.5 | 38.2 | 17.4 | 29.1 |
| New Zealand | 12.4 | 16.8 | 9.6 | 13.6 |
| Taiwan | 14.1 | 16.9 | 5.5 | 10.0 |
| United Kingdom | 7.3 | 9.46* | 5.5 | 7.23* |
| United States | 34.3 | 42.7 | 25.9 | 39.2 |

^6^Data extracted from the the Organisation for Economic Co-operation and Development and Policy Research Indicators Database
